# The Effects of Exendin-4 Treatment on Graft Failure: An Animal Study Using a Novel Re-Vascularized Minimal Human Islet Transplant Model

**DOI:** 10.1371/journal.pone.0121204

**Published:** 2015-03-20

**Authors:** Afaf Sahraoui, Maria Sörhede Winzell, Tracy Gorman, Dave M. Smith, Stanko Skrtic, Merete Hoeyem, Shadab Abadpour, Lars Johansson, Olle Korsgren, Aksel Foss, Hanne Scholz

**Affiliations:** 1 Institute for Surgical Research and Section for Transplantation Surgery, Oslo University Hospital, Oslo, Norway; 2 AstraZeneca R&D, Mölndal, Sweden; 3 AstraZeneca, Alderley Park, Cheshire, United Kingdom; 4 Department of Radiology, Oncology and Radiation Sciences, Uppsala University Hospital, Uppsala, Sweden; 5 Department of Immunology, Genetics and Pathology, Science for Life Laboratory, Uppsala University, Uppsala, Sweden; 6 Institute of Clinical Medicine, University of Oslo, Oslo, Norway; La Jolla Institute for Allergy and Immunology, UNITED STATES

## Abstract

Islet transplantation has become a viable clinical treatment, but is still compromised by long-term graft failure. Exendin-4, a glucagon-like peptide 1 receptor agonist, has in clinical studies been shown to improve insulin secretion in islet transplanted patients. However, little is known about the effect of exendin-4 on other metabolic parameters. We therefore aimed to determine what influence exendin-4 would have on revascularized minimal human islet grafts in a state of graft failure in terms of glucose metabolism, body weight, lipid levels and graft survival. Introducing the bilateral, subcapsular islet transplantation model, we first transplanted diabetic mice with a murine graft under the left kidney capsule sufficient to restore normoglycemia. After a convalescent period, we performed a second transplantation under the right kidney capsule with a minimal human islet graft and allowed for a second recovery. We then performed a left-sided nephrectomy, and immediately started treatment with exendin-4 with a low (20μg/kg/day) or high (200μg/kg/day) dose, or saline subcutaneously twice daily for 15 days. Blood was sampled, blood glucose and body weight monitored. The transplanted human islet grafts were collected at study end point and analyzed. We found that exendin-4 exerts its effect on failing human islet grafts in a bell-shaped dose-response curve. Both doses of exendin-4 equally and significantly reduced blood glucose. Glucagon-like peptide 1 (GLP-1), C-peptide and pro-insulin were conversely increased. In the course of the treatment, body weight and cholesterol levels were not affected. However, immunohistochemistry revealed an increase in beta cell nuclei count and reduced TUNEL staining only in the group treated with a low dose of exendin-4 compared to the high dose and control. Collectively, these results suggest that exendin-4 has a potential rescue effect on failing, revascularized human islets in terms of lowering blood glucose, maintaining beta cell numbers, and improving metabolic parameters during hyperglycemic stress.

## Introduction

Transplantation of insulin producing beta cells, or islets of Langerhans, can reverse hyperglycemia, and has become an alternative to insulin replacement therapy for selected patients with type 1 diabetes. However, more than one donor pancreas will often be required in order to restore normoglycemia, though the procedure has improved vastly over the last decade with a 5 year graft survival rate exceeding 50% [1,2]. This improvement is due to donor selection, islet isolation, T-cell depleting induction therapy, and tailored maintenance immunosuppression [2,3]. Still, islet transplantation is hampered by low long-term graft survival, especially with single donor transplantations, on account of several contributing variables [4]. Therefore, research that can improve single donor graft survival will be of great impact since available donor organs are scarce [5]. In this respect, the incretins, such as GLP-1, have been remarked upon as promising pharmacological interventions due to their ability to regulate glucose metabolism [6]. Exendin-4, an incretin mimetic (GLP-1 receptor agonist), has in numerous rodent models been shown to regulate blood glucose, stimulate beta cell neogenesis [7] and reduce apoptosis [8,9], thereby increasing beta cell mass. It is debated whether exendin-4 has such a potential effect on human beta cells, even though such reports do exist [10,11].

In this study, we aimed to investigate the effect of exendin-4 on engrafted, revascularized islets that were in a state of graft failure due to hyperglycemic stress. We therefore developed a novel animal transplant model where we introduce bilateral, subcapsular islet transplantations. This model allowed us to transplant diabetic mice with murine islet grafts sufficient enough to restore euglycemia before a minimal human islet graft was transplanted. In this way, we ensured revascularization before the murine graft was removed causing hyperglycemic stress to the remaining minimal human islet graft, and thereby mimicking graft failure. Simultaneously, we intervened with exendin-4 treatment, and evaluated the effects on the graft by measuring relevant metabolic parameters.

## Material and Methods

### Animal welfare

Animal experiments were approved by the Norwegian Animal Research Committee (Oslo, Norway) (permit number Id-3458) and conform to the Guide for the Care and Use of Laboratory Animals published by the US National Institutes of Health (NIH Publication, 8th Edition, 2011), and to the Norwegian Animal Welfare Act.

The animals were housed no more than 5 mice per cage. They were maintained in a 12h light/dark cycle in an approved unit and given free access to food and water except when fasting. The mice were handled by an experienced animal technician at all times, and all efforts were made to minimize suffering. The same technician monitored animal welfare in accordance to standardized requirements for the animal unit at Oslo University Hospital, administered the treatment, and performed the blood sampling.

### Murine islet isolation

Mouse islets were isolated from 8–10 weeks old male Balb/c mice (Taconic, Denmark) as previously described [12]. Briefly, 3.0 ml of Hank’s balanced salt solution containing 8mg/ml Collagenase P from Clostridium histolyticum (Roche, Mannheim, Germany) was injected into the pancreatic duct. The distended pancreas was subsequently removed and incubated at 37°C for 17 min followed by gradient purification of the endocrine tissue. The islets were placed in 90-mm-Petri dishes (Sterilin, Heger AS, Norway), and cultured overnight in RPMI 1640 media without L-glutamine (HyClone, Utah, USA) supplemented with 10% heat-inactivated fetal bovine serum, 1% penicillin/streptomycin, 10 mM 5ml Hepes and 1% L-glutamine (Gibco, Paisley, UK) at 37°C (5% CO_2_). The transplantation was performed the next day as described below.

### Human islet isolation and culture

Human islets were isolated using a modified semi-automated digestion method [13] from a male and female deceased donor aged 39 and60 years provided by the islet isolation facility of the Nordic Network in Uppsala, Sweden, after appropriate consent was given for multi-organ donation. The Regional Committee for Medical and Health Research Ethics Central in Norway approved the use of human islets for research (permit number 2011/426a).

Islet purity ranged between 70% and 90% as judged by dithizone staining, but the islet preparations were disqualified for clinical transplantation due to quantitative insufficiency. Islets were obtained within 3 days of isolation, and aliquots of the islet preparations were placed in 90-mm-Petri dishes (Sterilin, Heger AS, Norway) and cultured overnight in CMRL 1066 (Invitrogen, Netherlands) containing 10% ABO-compatible human serum, 1% penicillin/streptomycin, 10 mM 5ml Hepes and 1% L-glutamine (Gibco, Paisley, UK) at 37°C (5% CO_2_) before transplantation was performed as described below.

### Study design (Fig. 1)

**Fig 1:**
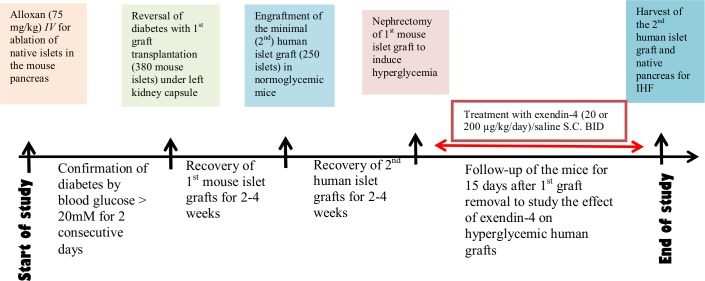
Time frame of experimental design. In the double islet transplantation model, the first mouse islet graft is used to achieve normoglycemia in diabetic mice. The second minimal human islet graft is allowed a convalescent period before the first mouse graft is removed to induce hyperglycemia (red arrow), and treatment -/+ exendin-4 with a low (20μg/kg/day) or high (200μg/kg/day) dose was initialized.

Male NMRI nu/nu immunodeficient mice (Taconic, Denmark), 8–10 weeks old, were used as recipients after being rendered diabetic by an intravenous infusion of Alloxan monohydrate (75 mg/kg, Sigma Aldrich, UK). Before transplantation, all recipients had non-fasting blood glucose levels ≥ 20 mmol/L for 2 consecutive days measured by a glucometer (Accu-Chek Aviva Nano, Roche Diagnostics, Indiana, USA). The mice were anesthetized with 1.5% Isoflurane (Baxter) mixed with oxygen, and given pre-operative analgesia with Buprenorphine (Temgesic, 0.1 mg/kg subcutaneously). 380 mouse islets were loaded into PE 50 tubing and then transplanted under the left kidney capsule of each mouse. The islet grafts were allowed to recover for 2–4 weeks, and function was assessed by monitoring non-fasting blood glucose levels twice a week. Mice with 2 consecutive measurements of non-fasting blood glucose levels > 11mmol/L, were excluded from the study. After recovery, a minimal mass islet graft of 250 human islets, handpicked, consistent in size and appearance as judged in a microscope, were transplanted under the right kidney capsule of each mouse previously transplanted with a mouse islet graft under the left kidney capsule. The scientist who picked the islets was blinded in regards to what mouse received which aliquot of beta cells. Islets from each donor were distributed systematically across the experimental groups to control for potential differences in islet quality from different donors. This was done by the animal technician. Eight mice were transplanted per donor per condition in two separate experiments. The transplantations were performed by the scientist. Following a new recovery period of 2 weeks, left-sided nephrectomy was performed taking out the first mouse islet graft, and treatment started with exendin-4 (Bachem, Bubendorf, Switzerland) at a dose of 20μg/kg/day (low) or 200μg/kg/day (high), and volume-matching saline as control, administered subcutaneously twice daily for 15 days. During follow-up, body weight, and non-fasting blood glucose levels were measured every third day at 8 a.m. prior to the administration of exendin-4. Fasting blood samples were taken at 7 and 15 days of treatment for analysis of metabolic parameters as described below. The mice were fasted for 4h after exendin-4 was administered. Only mice with blood glucose between 11–20 mmol/L were included in the study after the left-sided nephrectomy in order to mimic graft failure.

### Oral glucose tolerance test (OGTT)

After 13 days of treatment with exendin-4, an OGTT was performed after an overnight fast where the animals were given free access to water. Healthy, untreated mice (same strain, gender and age as study animals) were included as normal controls to provide a baseline. The test was performed 30 min after exendin-4 was administered or not. Each mouse was gavaged with 1.5 g/kg D-glucose (Fresenius Kabi, Oslo, Norway). Blood glucose was measured at 0, 15, 30, 60 and 120 min after glucose administration using a glucometer (Accu-Chek Aviva Nano, Roche Diagnostics, Indiana, USA). The area under the curve (AUC) of the glucose levels was calculated for each mouse.

### Blood samples and analysis of metabolic parameters

Blood samples were taken from the saphenous vein after 7 days of treatment, and at the end of the follow-up period, the animals were sacrificed and blood was collected by heart puncture into EDTA-coated or serum tubes (Becton, Dickinson and Company, Puls A/S, Oslo, Norway). The mice were injected subcutaneously with a mixture of Midazolam (Dormicum) and Fentanyl (Hypnorm) for sedation and analgesia before sacrifice was performed with dislocation of the neck. Collected samples were separated by centrifugation at 3000 rpm for 15 min and stored at -70°C awaiting assessment. Plasma human C-peptide, active GLP-1 (i.e. GLP-1 (7–36) amide) and glucagon were measured using a diabetes 4-plex assay (Bio-Plex, Bio-Rad Laboratories, Hercules, CA) analyzed on a Multiplex Analyzer (Bio-Rad Laboratories) following the manufacturer’s description. Circulating human pro-insulin levels were measured in serum samples using an ELISA assay according to the manufacturer’s description (Mercodia AB, Uppsala, Sweden). Serum triglycerides and total cholesterol concentrations were measured using enzymatic colorimetric reagents (Wako, Osaka, Japan) according to the manufacturer’s description.

### Immunofluorescent detection and evaluation of beta cell mass

For immunofluorescence, the transplanted human islet grafts were harvested, fixed and paraffin-embedded within 24 h of removal before deparaffinised using xylene and dehydrated in a graded series of ethanol washes (100%, 85%, 70%, and 50%). Tissue sections (4 μm) were subjected to epitope retrieval in 1X Target Retrieval Solution (DAKO, Denmark) in a hot water bath (99°C) for 20 min. Slides were then incubated with 0.2% Triton-X 100 in PBS for 30 min followed by incubation with Protein Block (serum-free) Reagent (DAKO, Denmark) in order to block non-specific staining. Slides were then incubated with polyclonal guinea pig anti-insulin 1:500 (DAKO, Denmark) overnight. After washing with Tris buffered saline and 0.05% (vol. /vol.) Tween (TBST), sections were incubated with secondary insulin antibodies (goat-anti-guinea pig AlexaFluor 488 for quantifying the beta cell numbers or AlexaFluor 594 for co-staining with TUNEL at the concentration of 1:300, Life Technologies AS, Oslo, Norway) for 1h at room temperature. To quantify the beta cell number, an automated imaging of the sections was carried out with the SpGreen filter on a Zeiss Axio Imager M1 (Carl Zeiss, Jena, Germany) operated through the Metafer software (MetaSystems, Waltham, MA, USA). A custom designed Metafer software classifier enabled mapping of all beta cells. Definiens image analysis software (Munich, Germany) was used to quantify all fluorescent images. Following insulin staining, apoptosis was measured by TUNEL staining using DeadEnd Fluorometric TUNEL system according to the manufacturer’s protocol (Promega Biotech AB, Sweden). Nuclear staining was performed using Slow Fade Gold antifade reagent with DAPI (Life Technologies AS, Oslo, Norway). Images were taken using the Axio Observer inverted microscope (Carl Zeiss AG, Germany) with the ZEN lite software. All the pictures were quantified and analyzed for apoptosis using Image J software. A minimum of 5000 cells per group were scored.

### Statistical analysis and calculations

All data are presented as means ±SD and the GraphPad Prism 6.0 statistical software (La Jolla, CA, USA) was used for data analysis. Differences between groups were evaluated by one-way ANOVA followed by Bonferroni correction. Significance was set at P < 0.05. The results of the metabolic parameters are stated as ∆, calculating the difference in outcome between day 15 and day 7 after treatment was initiated.

## Results

### Exendin-4 improves the random blood glucose levels in diabetic mice transplanted with minimal human islet grafts

**Fig 2:**
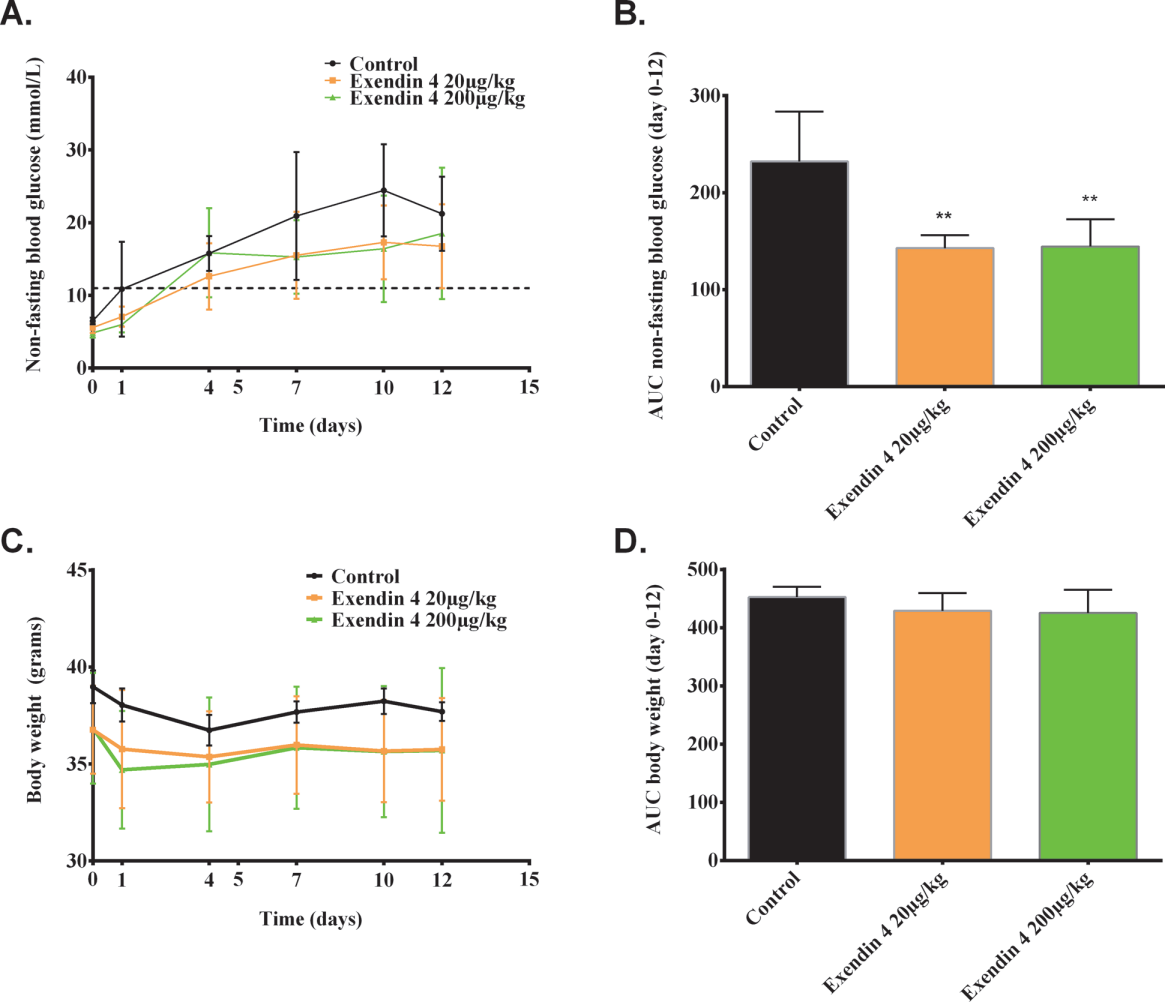
Exendin-4 treatment improves non-fasting blood glucose regardless of dose. Mice were transplanted with a minimal human islet graft (250 islets) and treated with or without a low (20μg/kg/day) or high (200μg/kg/day) dose of exendin-4 for 15 days while monitoring glycemic levels and body weight for saline treated controls (black line), and exendin-4 treated groups (20μg/kg/day (orange line) and 200μg/kg/day (green line)). (a) Random blood glucose levels over the first 12 days of treatment (b) with corresponding AUC representing the different groups. (c) Body weight during the first 12 days of treatment (d) with corresponding AUC calculated for each group. **p<0.01 by one-way ANOVA followed by Bonferroni correction vs. corresponding saline treated control, and values are expressed as mean ±SD.

Non-fasting blood glucose levels were significantly reduced following 15 days of exendin-4 treatment (20 and 200 μg/kg/day) compared to saline treated control (p<0.01). This effect is independent of the exendin-4 dose (Fig. 2B). The body weight did not differ among the groups during the 15 days of treatment with either low (20μg/kg/day) or high (200μg/kg/day) doses of exendin-4 compared to the saline treated control (Fig. 2C and 2D).

### The glucose tolerance in exendin-4 treated hyperglycemic mice is improved after an acute administration of exendin-4

**Fig 3:**
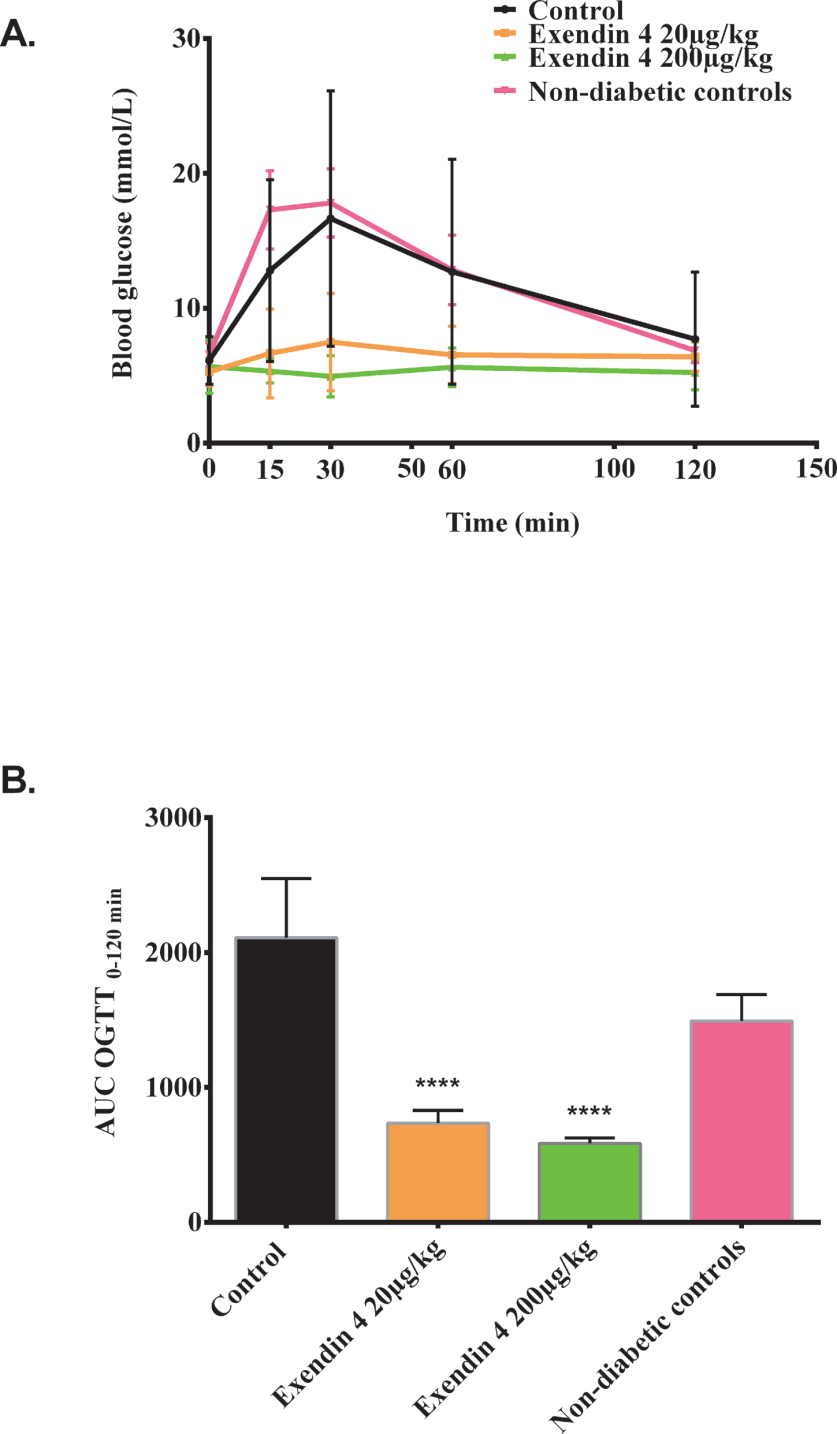
Acute administration of exendin-4 improves the oral glucose tolerance test. Mice transplanted with a minimal human islet graft (250 islets) were treated with or without exendin-4 (20 or 200μg/kg/day) for 15 days. At day 13 day of +/-exendin-4 treatment, an oral glucose tolerance test was performed as indicated in methods. The control group (black line) was compared to non-diabetic controls (pink line), and exendin-4 treated groups (20μg/kg/day (orange line), 200μg/kg/day (green line)). (a) Blood glucose levels during the oral glucose tolerance test with (b) AUC calculated per mice in the different groups. Results are expressed as mean ±SD for each column. **** p< 0.0001 by one-way ANOVA followed by Bonferroni correction vs. saline treated control.

The GLP-1 receptor agonist exendin-4 regulates blood glucose by enhancing glucose-dependent insulin secretion and decreasing gastric emptying [14]. To examine the effect of exendin-4 on the glucose sensitivity of diabetic mice transplanted with minimal human islet grafts, an OGTT was performed after 13 days of treatment (Fig. 3). We hypothesized that the exendin-4 treated groups would have lower blood glucose than the saline treated control and the non-diabetic mice. Additionally, we speculated that the greatest reduction in hyperglycemia would be in the group treated with the highest dose (200μg/kg/day) of exendin-4 cf Arakawa et al [15]. We find that a single administration of a low or high dose of exendin-4 30 min prior to the OGTT, significantly improved the glucose tolerance equally in these animals (Fig. 3A), which was also confirmed by reduced glucose excursion visualized as AUC measurements (Fig. 3B) (p<0.0001).

### Exendin-4 increased human C-peptide levels in diabetic mice transplanted with minimal human islet grafts

**Fig 4:**
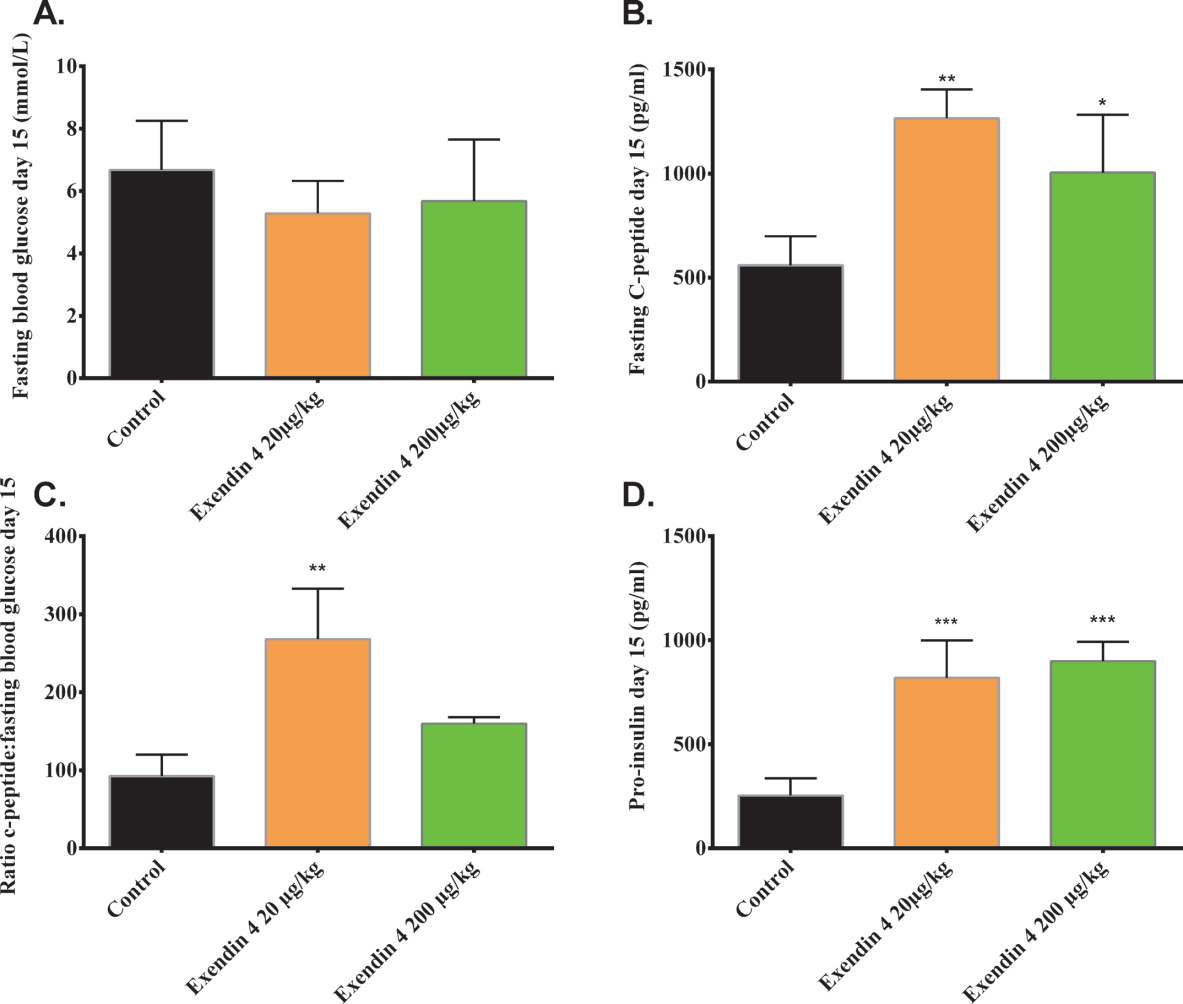
Exendin-4 treatment increases C-peptide and pro-insulin secretion in mice transplanted with minimal human islet grafts. Circulating levels of fasting blood glucose (a) and C-peptide (b) after 15 days of treatment -/+ exendin-4 (20 or 200μg/kg/day). (c) Ratio of fasting C-peptide to fasting blood glucose at day 15 of treatment was calculated for each mouse. (d) Pro-insulin was measured as indicated in methods. Values for each column are expressed as mean ±SD. * p<0, 05, ** p <0, 01, *** p< 0, 001 by one-way ANOVA followed by Bonferroni correction vs. saline treated control.

Fasting blood glucose levels in diabetic mice transplanted with minimal human islet grafts were similar after 15 days of treatment ± exendin-4 (Fig. 4A). Human plasma C-peptide levels, however, were significantly increased for both doses (20μg/kg/day and 200μg/kg/day; p<0.01 and p<0.05) (Fig. 4B). The fasting blood glucose levels after 15 days of treatment (Fig. 4A) were: control 6.7 ± 1.6 mmol/L vs. exendin-4 low (20μg/kg/day) 5.3 ± 1.0 mmol/L vs. exendin-4 high (200μg/kg/day) 5.7 ±2.0 mmol/L. The corresponding levels of C-peptide were: control 559 ± 139 pg/ml vs. exendin-4 (20μg/kg/day) 1265± 138 pg/ml vs. exendin-4 (200μg/kg/day) 1005 ± 277 pg/ml (Fig. 4B). As an index for beta cell function, the ratio of C-peptide to fasting blood glucose after 15 days of treatment -/+ exendin-4 was calculated. A significantly increased ratio (p<0.01) in the group treated with a low (20μg/kg/day) dose compared to control (Fig. 4C) was found. After 15 days of treatment, serum human pro-insulin levels were significantly higher in mice treated with exendin-4 at a dose of either 20μg/kg/day (819 ± 180 pg/ml) or 200μg/kg/day (900 ± 93 pg/ml), p<0.001) compared to untreated control (254 ± 82 pg/ml) (Fig. 4D).

### Exendin-4 treatment affects metabolic parameters in diabetic mice transplanted with minimal human islet grafts

**Fig 5:**
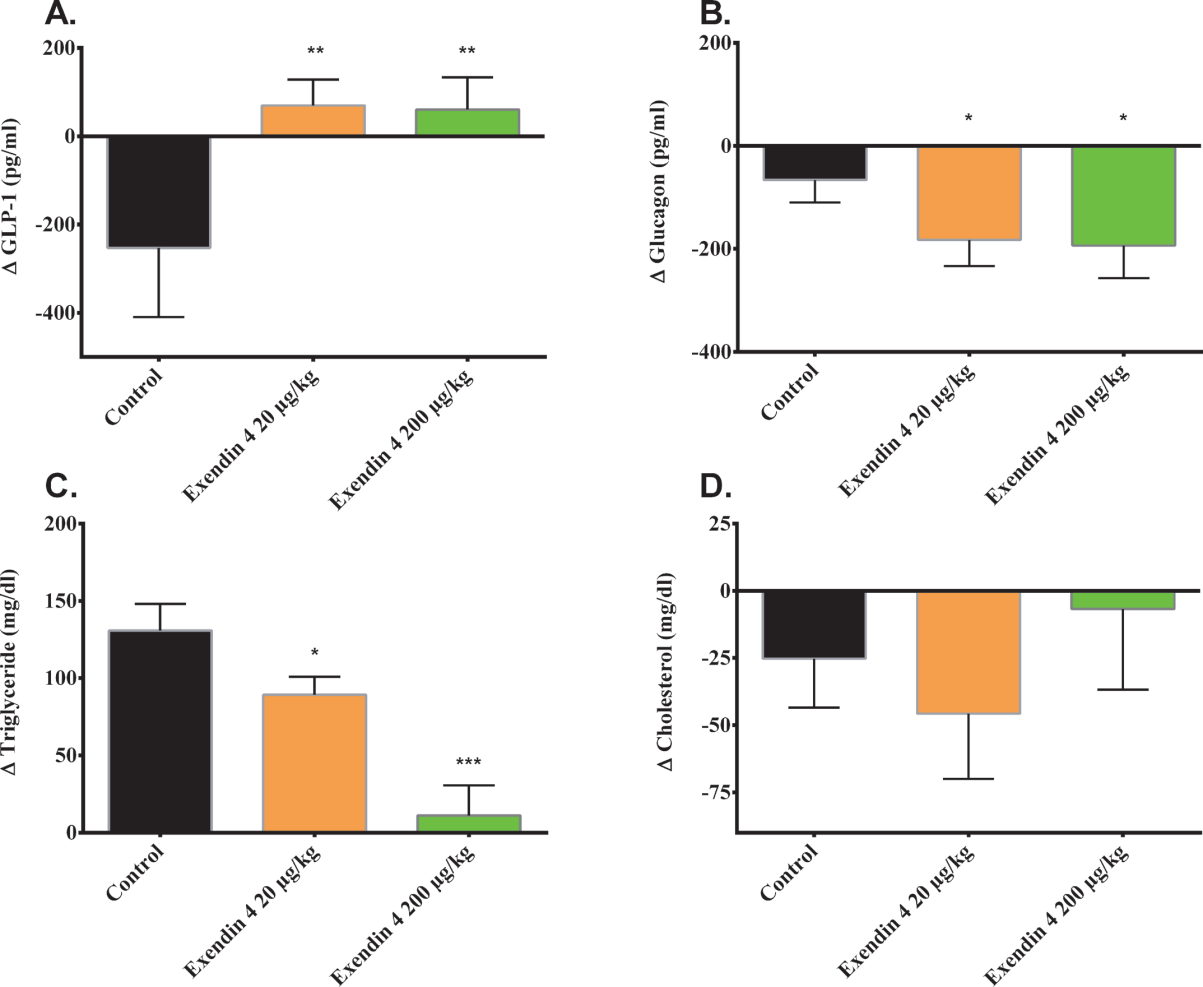
Exendin-4 treatment improves endogenous GLP-1 production, reduces glucagon release and improves triglyceride profile. Blood sampling was performed on day 7 and 15 of treatment -/+ exendin-4 with a low (20μg/kg/day) or high (200μg/kg/day) dose. Analysis of selected metabolic parameters was performed and the difference (∆) between day 7 and 15 was calculated for each mouse as indicated in methods for (a) GLP-1, (b) glucagon, (c) triglycerides and (d) total cholesterol. Results are expressed as mean ±SD. * p<0, 05, ** p<0, 01, *** p<0, 001 by one-way ANOVA followed by Bonferroni correction vs. saline treated control.

We tested whether the improvement of human islet function after exendin-4 treatment, indicated by increased C-peptide levels, could also affect other metabolic parameters. Active GLP-1 levels in mice were significantly increased after 15 days treatment with exendin-4 compared to untreated control (Fig. 5A, p<0.01). Plasma glucagon concentration was equally reduced in mice treated with exendin-4 at a dose of 20 and 200 μg/kg/day compared to the untreated control (Fig. 5B, p<0.05). Exendin-4 treatment significantly reduced the serum levels of triglycerides (Fig. 5C) at the dose of 20μg/kg/day (p<0.05), and almost abolished the levels at the dose of 200μg/kg/day (p<0.001) compared to untreated control. However, exendin-4 had no effect on serum cholesterol levels which was comparable among the groups (Fig. 5D) and did not significantly differ.

### Higher beta cell numbers in exendin-4 treated diabetic mice transplanted with minimal human islet grafts

**Fig 6:**
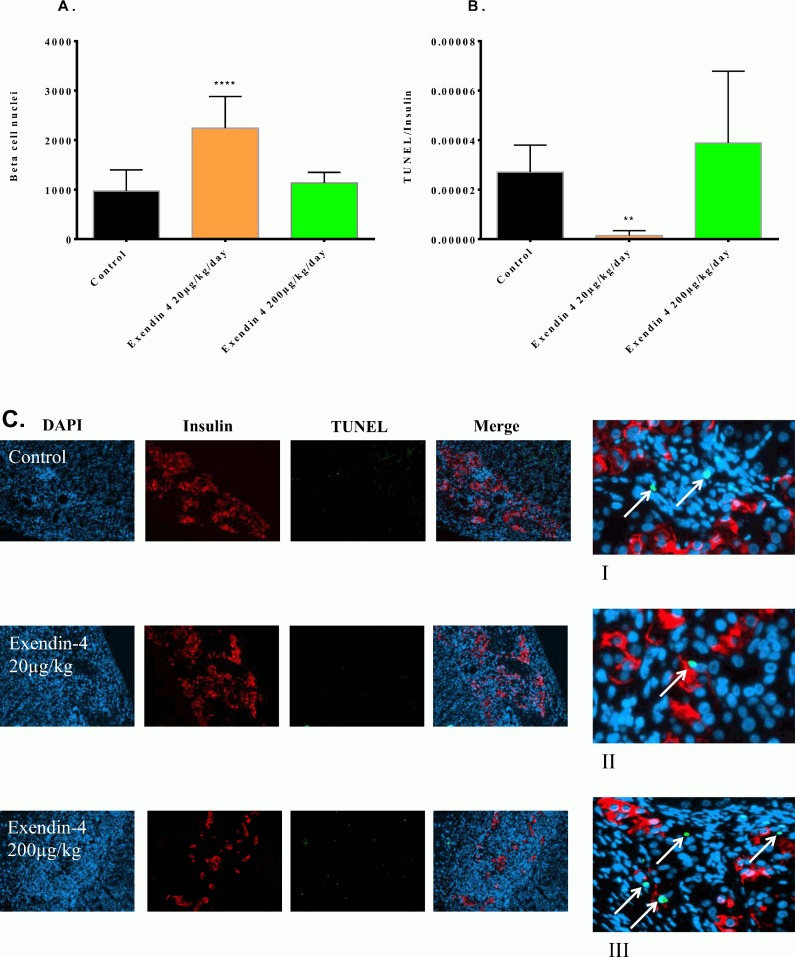
Exendin-4 treatment increases transplanted human beta cell mass, and reduces apoptosis. After 15 days of treatment -/+ exendin-4 treatment with a low (20μg/kg/day) or high (200μg/kg/day) dose, the cell nuclei in the grafts were counted (a). The ratio of TUNEL positive cells to insulin was calculated in the transplanted grafts after 15 days of exendin-4 treatment. Immunofluorescence staining was performed as indicated in methods, and representative images (c) show DAPI (blue), insulin (red) and TUNEL (green) staining in transplanted human islets treated with a low (20μg/kg/day) or high (200μg/kg/day) dose of exendin-4, or saline (control). The right-hand panel (I-III) shows TUNEL staining indicated by the arrows. The results for each column are expressed as mean ±SD. **** p<0, 0001 by one-way ANOVA followed by Bonferroni correction vs. saline treated control.

In rodent islets, studies on exendin-4 have shown a potential for inducing beta cell replication [10,15], or even neogenesis [7]. In human islets it is debated whether such a potential is realistic, though there have been studies that may have shown increased beta cell replication [10] and neogenesis [16] in human beta cell grafts transplanted to mouse recipients. We therefore wanted to quantify if exendin-4 could preserve, or even increase the minimal mass human islet grafts that we had transplanted to recipients that were treated for 15 days with ±exendin-4. With immunohistochemical staining and quantification, we found a significant increase in the beta cell numbers (Fig. 6A) in the grafts subjected to a low dose (20μg/kg/day) exendin-4 treatment compared to untreated control (2240±641 vs.972±426 beta cell nuclei) (p<0.0001). However, no increase in the beta cell nuclei count in the group that was treated with a high dose (200μg/kg/day) of exendin-4 (1134±215 beta cell nuclei) was observed. Calculating the ratio of TUNEL, a marker for apoptosis, to insulin (Fig. 6B), a significant decrease in the group treated with a low (20μg/kg/day) dose of exendin-4 (p<0.01) was demonstrated while no such correlation was found in the high dose group. The immunohistofluorescence also shows more insulin and less TUNEL staining in this group (Fig. 6C, panel II) compared to the untreated control.

## Discussion

In this study, we introduce the double islet transplantation model as a method to investigate the effects of exendin-4 treatment on maintaining functional beta cell mass in *revascularized* minimal human islet grafts with partial function in the hyperglycemic state. This model was developed specifically to mimic the state of a failing, but vascularized islet graft. King et al [17] implemented a similar approach in their work, but they experimented on mouse islets, performed the transplantations simultaneously and started treatment with exendin-4 immediately post-operatively. They treated the mice in a normoglycemic state and the islet grafts were not revascularized when the treatment started. We therefore developed this model further in order to investigate whether graft dysfunction could be ameliorated by medical intervention with a GLP-1 receptor agonist, since this could be a realistic scenario for many islet transplanted patients. We compared a low (20μg/kg/day) and high (200μg/kg/day) dose treatment of exendin-4 to a saline treated group (control). The choice of treatment doses was based on our own experience with this compound, but also on what other studies have shown previously [10,18].

In this study we found that exendin-4 treatment reduced blood glucose and improved glucose tolerance. Also, during the course of 15 days’ treatment, exendin-4 increased C-peptide, pro-insulin, and active GLP-1. Glucagon and triglycerides were significantly decreased compared to the saline treated control group, but cholesterol was not affected. Additionally, we find that exendin-4 has a conserving effect on the beta cell mass during hyperglycemic stress.

The effect of exendin-4 has previously been studied in transplanted type 1 diabetic patients [19,20]. These studies reveal that exendin-4 stimulates insulin secretion in islet transplanted recipients. However, HbA_1_c remained unchanged by the exendin-4 treatment. This suggests that the effect of exendin-4 on blood glucose may only be temporary and could mask the inevitable deterioration of islet graft function. In both these studies, the patients were started on exendin-4 treatment after engraftment of the islets, and the focus revolved around glucose metabolism and insulin secretion. In this study, we also wished to investigate the effect exendin-4 has on other relevant parameters. We did not measure HbA1c in our mice given the short treatment period, but similar to the human subjects, exendin-4 stimulated insulin secretion in human islets transplanted to mice. An interesting finding was the apparent increase in beta cell nuclei count in the grafts treated with a low (20μg/kg/day) dose of exendin-4. The findings from the immunohistofluorescence analysis suggest that this is caused by a conserving effect of exendin-4 on the beta cell number. Reduced TUNEL staining suggests reduced apoptosis in the group treated with a low exendin-4 dose compared to the control. Accordingly, the apparent increase in beta cell number seems to be a consequence of an anti-apoptotic effect of exendin-4 protecting the transplanted graft which several studies have demonstrated [21–24], and our observations are consistent with these. Contradictory, we did not observe such a protective effect of exendin-4 when we administered the compound in a high dose (200μg/kg/day). There was no decrease in TUNEL/insulin ratio, and also no increase in beta cell nuclei count. This indicates that exendin-4 has a bell-shaped dose response curve in vivo, an observation consistent with what has been reported previously [25–27]. No mechanisms have been identified to explain this effect [28], however GLP-1 receptor saturation at high exendin-4 concentrations has been proposed [29]. The response is also matched by our other findings where we see similar improvements in metabolic parameters in the exendin-4 treated subjects, regardless of the dose. With the doses we've chosen, we see a similar response for C-peptide and pro-insulin after 15 days of exendin-4 treatment. We also find the same correlation for active GLP-1 and glucagon. The only parameter we find that benefits from the highest dose of exendin-4 (200μg/kg/day), are the triglycerides. Otherwise we find no additional benefit from administering the highest dose. Parlevliet et al [30] showed that exendin-4 reduces hepatic lipogenesis, which caused a reduction in hepatic triglyceride content, hence the reduction of circulating triglycerides. In the case of triglycerides, it could be that the bell-shaped response curve is displaced compared to the other parameters, and therefore the reason for why we measure no differences among the groups. However, in order to precisely answer this question, a dose-response study is needed. To our knowledge, no study has shown the effect of exendin-4 on the lipid profile in recipients transplanted with human islets. Although this effect has been observed in type 2 diabetics treated with exendin-4 [31], these patients were not transplanted.

In conclusion, we find a positive effect of exendin-4 on vascularized transplanted human islet in a state of graft failure, but this effect is dependent on the dose in a bell-shaped manner. The GLP-1 receptor agonist has valuable attributes in addition to its blood glucose lowering properties that could benefit islet transplanted patients. The anti-apoptotic effect that contributes to maintaining beta cell mass during graft failure is of particular interest. The effect on triglycerides is also desirable. Patients with diabetes have often metabolic dysregulation, and lowering the hepatic lipogenesis could be beneficial. Our data therefore support exendin-4 as a strategic intervention to improve graft survival in vascularized human islet grafts.

## Supporting Information

S1 ARRIVE Checklist Checklist for in vivo animal experiments. (DOCX)

## References

[1] Barton FB, Rickels MR, Alejandro R, Hering BJ, Wease S, Naziruddin B, et al (2012) Improvement in outcomes of clinical islet transplantation: 1999–2010. Diabetes Care 35: 1436–1445. 10.2337/dc12-0063 22723582 PMC3379615

[2] Shapiro AM (2011) State of the art of clinical islet transplantation and novel protocols of immunosuppression. Curr Diab Rep 11: 345–354. 10.1007/s11892-011-0217-8 21830042

[3] Berney T, Johnson PR (2010) Donor pancreata: evolving approaches to organ allocation for whole pancreas versus islet transplantation. Transplantation 90: 238–243. 10.1097/TP.0b013e3181e25a40 20463635

[4] Kanak MA, Takita M, Kunnathodi F, Lawrence MC, Levy MF, Naziruddin B (2014) Inflammatory response in islet transplantation. Int J Endocrinol 2014: 451035 10.1155/2014/451035 24883060 PMC4021753

[5] Al-Adra DP, Gill RS, Imes S, O'Gorman D, Kin T, Axford SJ, et al. (2014) Single-Donor Islet Transplantation and Long-term Insulin Independence in Select Patients With Type 1 Diabetes Mellitus. Transplantation. 10.1097/TP.0000000000000217 24911037

[6] Drucker DJ, Nauck MA (2006) The incretin system: glucagon-like peptide-1 receptor agonists and dipeptidyl peptidase-4 inhibitors in type 2 diabetes. Lancet 368: 1696–1705. 17098089 10.1016/S0140-6736(06)69705-5

[7] Kwon DY, Kim YS, Ahn IS, Kim da S, Kang S, Hong SM, et al (2009) Exendin-4 potentiates insulinotropic action partly via increasing beta-cell proliferation and neogenesis and decreasing apoptosis in association with the attenuation of endoplasmic reticulum stress in islets of diabetic rats. J Pharmacol Sci 111: 361–371. 20019445 10.1254/jphs.09178fp

[8] Broca C, Varin E, Armanet M, Tourrel-Cuzin C, Bosco D, Dalle S, et al (2014) Proteasome dysfunction mediates high glucose-induced apoptosis in rodent beta cells and human islets. PLoS One 9: e92066 10.1371/journal.pone.0092066 24642635 PMC3958412

[9] Farilla L, Bulotta A, Hirshberg B, Li Calzi S, Khoury N, Noushmehr H, et al (2003) Glucagon-like peptide 1 inhibits cell apoptosis and improves glucose responsiveness of freshly isolated human islets. Endocrinology 144: 5149–5158. 12960095 10.1210/en.2003-0323

[10] Tian L, Gao J, Weng G, Yi H, Tian B, O'Brien TD, et al (2011) Comparison of exendin-4 on beta-cell replication in mouse and human islet grafts. Transpl Int 24: 856–864. 10.1111/j.1432-2277.2011.01275.x 21627696

[11] Xu G, Stoffers DA, Habener JF, Bonner-Weir S (1999) Exendin-4 stimulates both beta-cell replication and neogenesis, resulting in increased beta-cell mass and improved glucose tolerance in diabetic rats. Diabetes 48: 2270–2276. 10580413 10.2337/diabetes.48.12.2270

[12] Szot GL, Koudria P, Bluestone JA (2007) Murine pancreatic islet isolation. J Vis Exp: 255. 10.3791/255 PMC2565847 18989427

[13] Goto M, Eich TM, Felldin M, Foss A, Kallen R, Salmela K, et al (2004) Refinement of the automated method for human islet isolation and presentation of a closed system for in vitro islet culture. Transplantation 78: 1367–1375. 15548977 10.1097/01.tp.0000140882.53773.dc

[14] Nadkarni P, Chepurny OG, Holz GG (2014) Regulation of glucose homeostasis by GLP-1. Prog Mol Biol Transl Sci 121: 23–65. 10.1016/B978-0-12-800101-1.00002-8 24373234 PMC4159612

[15] Arakawa M, Ebato C, Mita T, Hirose T, Kawamori R, Fujitani Y, et al (2009) Effects of exendin-4 on glucose tolerance, insulin secretion, and beta-cell proliferation depend on treatment dose, treatment duration and meal contents. Biochem Biophys Res Commun 390: 809–814. 10.1016/j.bbrc.2009.10.054 19836346

[16] Suarez-Pinzon WL, Rabinovitch A (2011) Combination therapy with a dipeptidyl peptidase-4 inhibitor and a proton pump inhibitor induces beta-cell neogenesis from adult human pancreatic duct cells implanted in immunodeficient mice. Cell Transplant 20: 1343–1349. 10.3727/096368910X557263 21396168

[17] King A, Lock J, Xu G, Bonner-Weir S, Weir GC (2005) Islet transplantation outcomes in mice are better with fresh islets and exendin-4 treatment. Diabetologia 48: 2074–2079. 16132945 10.1007/s00125-005-1922-0

[18] Luo J, Nguyen K, Chen M, Tran T, Hao J, Tian B, et al (2013) Evaluating insulin secretagogues in a humanized mouse model with functional human islets. Metabolism 62: 90–99. 10.1016/j.metabol.2012.07.010 22982177

[19] Faradji RN, Froud T, Messinger S, Monroy K, Pileggi A, Mineo D, et al (2009) Long-term metabolic and hormonal effects of exenatide on islet transplant recipients with allograft dysfunction. Cell Transplant 18: 1247–1259. 10.3727/096368909X474456 20003758

[20] Froud T, Faradji RN, Pileggi A, Messinger S, Baidal DA, Ponte GM, et al (2008) The use of exenatide in islet transplant recipients with chronic allograft dysfunction: safety, efficacy, and metabolic effects. Transplantation 86: 36–45. 10.1097/TP.0b013e31817c4ab3 18622276 PMC2772201

[21] Chen J, Couto FM, Minn AH, Shalev A (2006) Exenatide inhibits beta-cell apoptosis by decreasing thioredoxin-interacting protein. Biochem Biophys Res Commun 346: 1067–1074. 16782054 10.1016/j.bbrc.2006.06.027

[22] Li L, El-Kholy W, Rhodes CJ, Brubaker PL (2005) Glucagon-like peptide-1 protects beta cells from cytokine-induced apoptosis and necrosis: role of protein kinase B. Diabetologia 48: 1339–1349. 15902400 10.1007/s00125-005-1787-2

[23] Li Y, Hansotia T, Yusta B, Ris F, Halban PA, Drucker DJ (2003) Glucagon-like peptide-1 receptor signaling modulates beta cell apoptosis. J Biol Chem 278: 471–478. 12409292 10.1074/jbc.M209423200

[24] Velmurugan K, Balamurugan AN, Loganathan G, Ahmad A, Hering BJ, Pugazhenthi S (2012) Antiapoptotic actions of exendin-4 against hypoxia and cytokines are augmented by CREB. Endocrinology 153: 1116–1128. 10.1210/en.2011-1895 22253425

[25] Fehmann HC, Habener JF (1991) Homologous desensitization of the insulinotropic glucagon-like peptide-I (7–37) receptor on insulinoma (HIT-T15) cells. Endocrinology 128: 2880–2888. 1645253 10.1210/endo-128-6-2880

[26] Goke R, McGregor GP, Goke B (1993) Amylin alters the biological action of the incretin hormone GLP-1(7–36)amide. Life Sci 53: 1367–1372. 7692203 10.1016/0024-3205(93)90597-v

[27] Kemp DM, Habener JF (2001) Insulinotropic hormone glucagon-like peptide 1 (GLP-1) activation of insulin gene promoter inhibited by p38 mitogen-activated protein kinase. Endocrinology 142: 1179–1187. 11181533 10.1210/endo.142.3.8026

[28] Parkes DG, Pittner R, Jodka C, Smith P, Young A (2001) Insulinotropic actions of exendin-4 and glucagon-like peptide-1 in vivo and in vitro. Metabolism 50: 583–589. 11319721 10.1053/meta.2001.22519

[29] Gao W, Jusko WJ (2012) Target-mediated pharmacokinetic and pharmacodynamic model of exendin-4 in rats, monkeys, and humans. Drug Metab Dispos 40: 990–997. 10.1124/dmd.111.042291 22338110 PMC3336795

[30] Parlevliet ET, Wang Y, Geerling JJ, Schroder-Van der Elst JP, Picha K, O'Neil K, et al (2012) GLP-1 receptor activation inhibits VLDL production and reverses hepatic steatosis by decreasing hepatic lipogenesis in high-fat-fed APOE*3-Leiden mice. PLoS One 7: e49152 10.1371/journal.pone.0049152 23133675 PMC3487842

[31] Nikfar S, Abdollahi M, Salari P (2012) The efficacy and tolerability of exenatide in comparison to placebo; a systematic review and meta-analysis of randomized clinical trials. J Pharm Pharm Sci 15: 1–30. 22365085 10.18433/j3g883

