# Supplementary material for: The Effects of Exendin-4 Treatment on Graft Failure: An Animal Study Using a Novel Re-Vascularized Minimal Human Islet Transplant Model
Source: PLoS One. 2015 Mar 20;10(3):e0121204. doi: 10.1371/journal.pone.0121204 (PMC4368803; doi:10.1371/journal.pone.0121204)

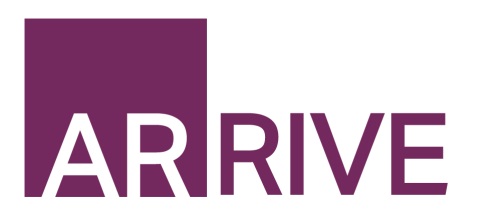


The ARRIVE Guidelines Checklist

Animal Research: Reporting In Vivo Experiments

Carol Kilkenny^1^, William J Browne^2^, Innes C Cuthill^3^, Michael Emerson^4^ and Douglas G Altman^5^

*^1^The National Centre for the Replacement, Refinement and Reduction of Animals in Research, London, UK, ^2^School of Veterinary Science, University of Bristol, Bristol, UK, ^3^School of Biological Sciences, University of Bristol, Bristol, UK, ^4^National Heart and Lung Institute, Imperial College London, UK, ^5^Centre for Statistics in Medicine, University of Oxford, Oxford, UK.*

|  | ITEM | RECOMMENDATION | Section/ Paragraph |  |
| --- | --- | --- | --- | --- |
| 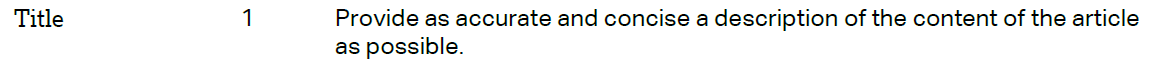 | | | | Title page |
| 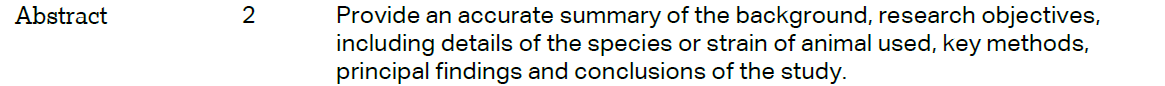 | | | | Page 2 |
| INTRODUCTION | | | |  |
| 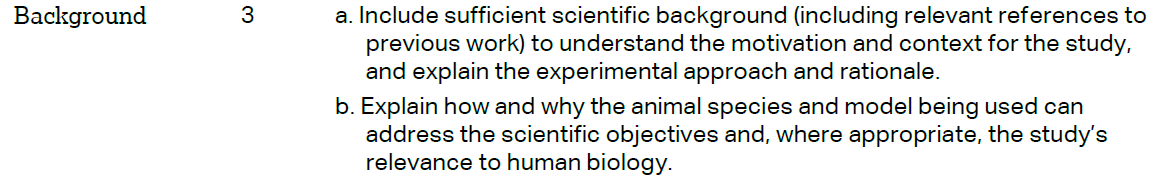 | | | | a and b are detailed under Introduction |
| 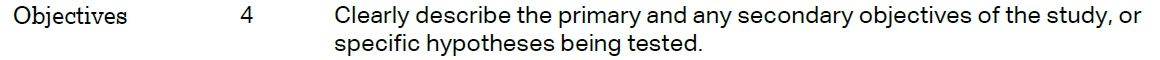 | | | | Introduction |
| METHODS | | | |  |
| 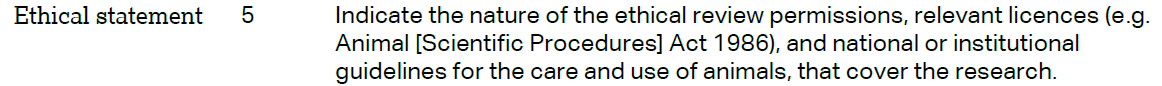 | | | | Methods, “Animal welfare” , paragraph 1 |
| 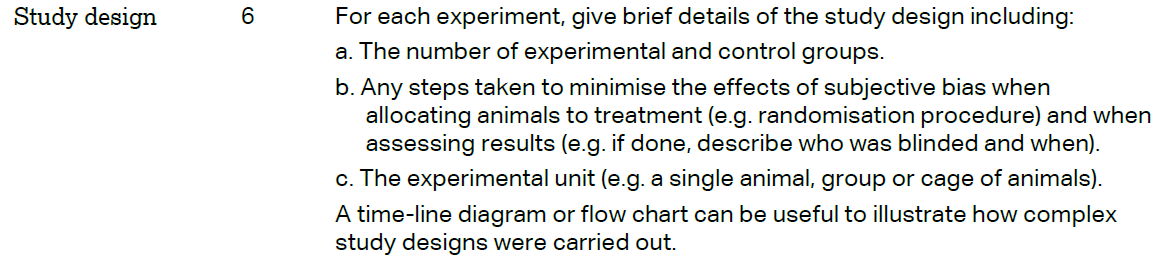 | | | | Methods:  a-c paragraph 4 |
| 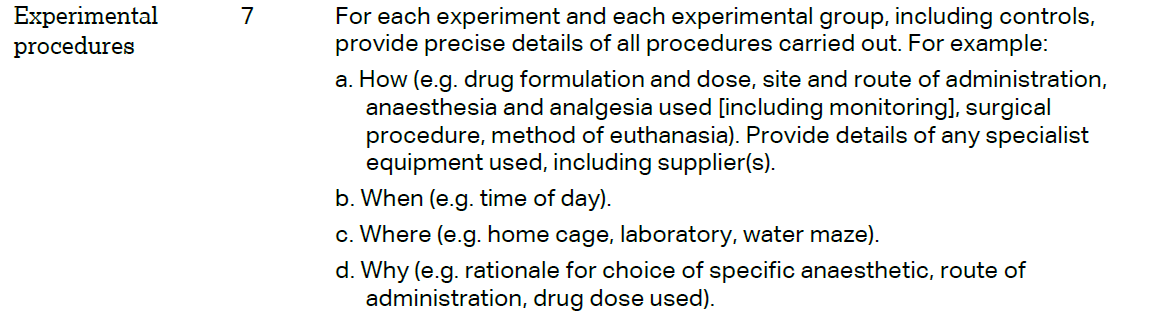 | | | | Methods: a-paragraph 1, 4, 5 and 6. b- paragraph 4 c-N/A d- Discussion: paragraph 1 |
| 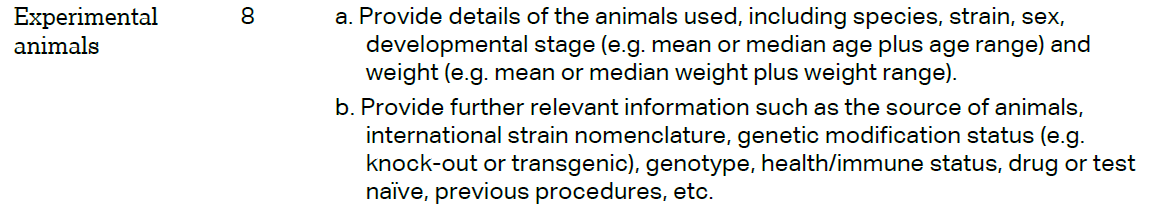 | | | | Methods: a and b-paragraph 2, and 4. |

The ARRIVE guidelines. Originally published in *PLoS Biology*, June 2010^1^

| 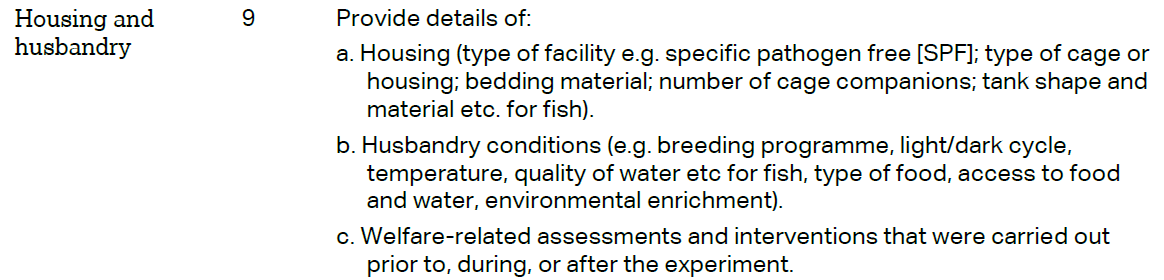 | Methods: a-c: paragraph 1 | |
| --- | --- | --- |
| 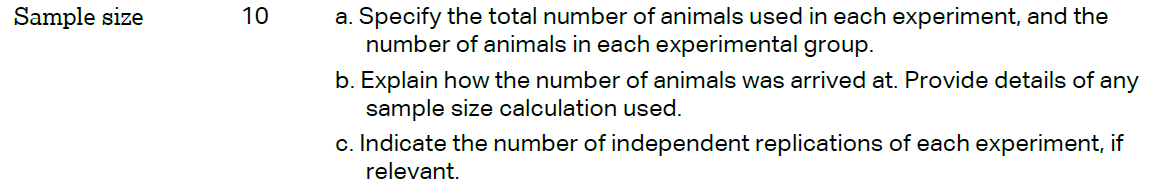 | Methods: a-paragraph 4. b-c: N/A | |
| 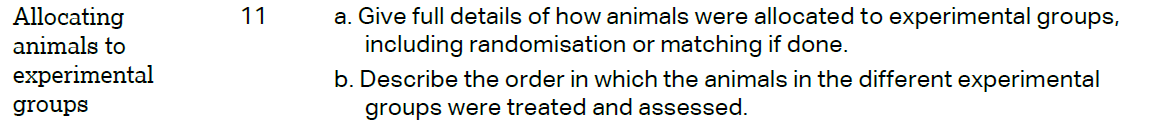 | Methods: a-paragraph | |
| 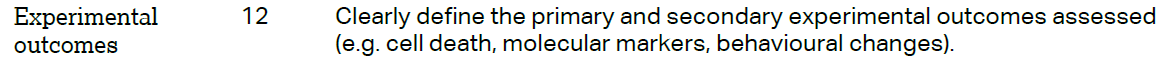 | Introduction | |
| 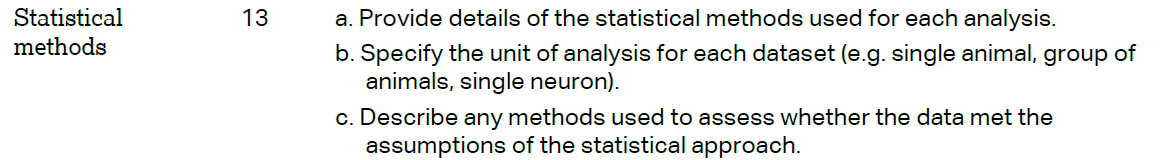 | Methods:  a-paragraph 8 b-paragraph 4  c- N/A | |
| RESULTS |  | |
| 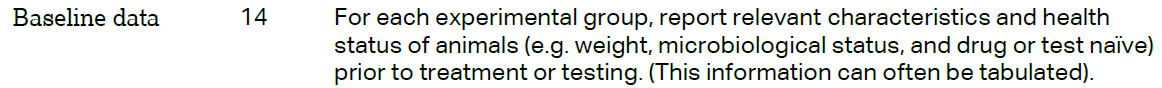 | Methods: paragraph 4 and 5 | |
| 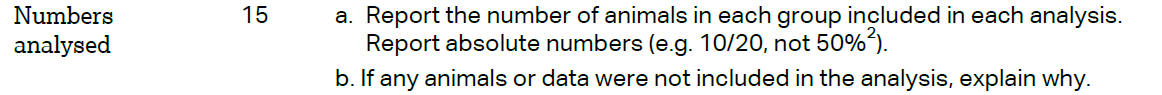 | Methods:  Paragraph 4 | |
| 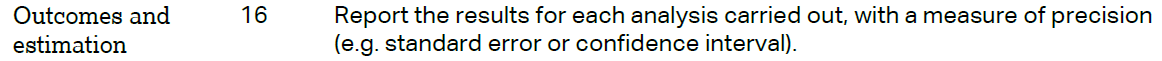 | Methods:  Paragraph 8 | |
| 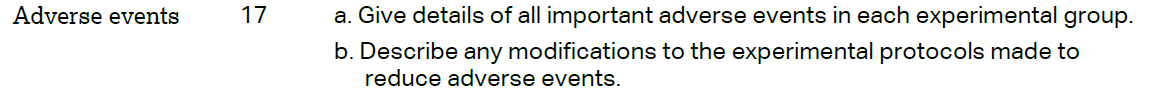 | N/A | |
| DISCUSSION |  | |
| 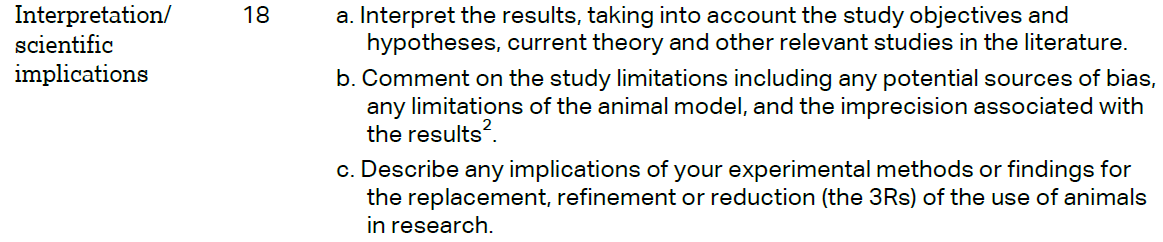 | Discussion a-b c-N/A | |
| 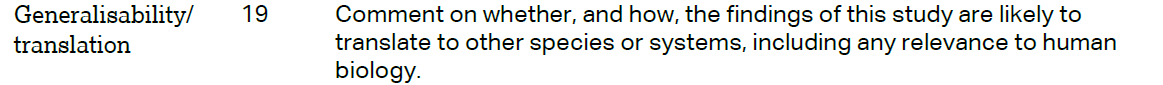 | Discussion | |
| 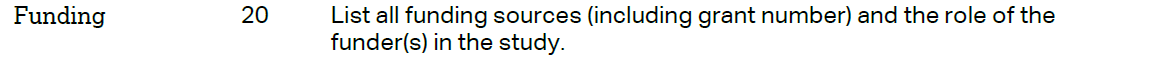 | | Submission form |


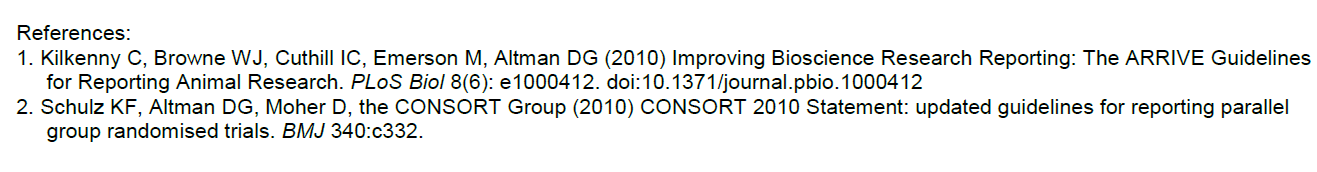

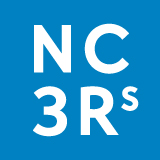

Supplement: S1 ARRIVE Checklist — (DOCX) [file pone.0121204.s001.docx]
